# Performance of Open-Source Large Language Models in Psychiatry: Usability Study Through Comparative Analysis of Non-English Records and English Translations

**DOI:** 10.2196/69857

**Published:** 2025-08-18

**Authors:** Min-Gyu Kim, Gyubeom Hwang, Junhyuk Chang, Seheon Chang, Hyun Woong Roh, Rae Woong Park

**Affiliations:** 1 Department of Biomedical Informatics, Ajou University School of Medicine, 206 World cup-ro, Yeongtong-gu, Suwon, 16499, Republic of Korea, 82 312194471, 82 312194472; 2 Center for Biomedical Informatics Research, Ajou University Medical Cencer, Suown, Republic of Korea; 3 Department of Medical Sciences, Graduate School of Ajou University, Suwon, Korea; 4 BK21 R&E Initiative for Advanced Precision Medicine, Suwon, Republic of Korea; 5 Department of Biomedical Sciences, Ajou University Graduate School of Medicine, Suwon, Republic of Korea; 6 Department of Computer Science, The University of Texas at Austin, Austin, TX, United States; 7 Department of Psychiatry, Ajou University School of Medicine, Suwon, Republic of Korea

**Keywords:** large language model, open-source, mental health, psychiatry, multilingual, translation, reasoning, diagnosis, accuracy, examination

## Abstract

**Background:**

Large language models (LLMs) have emerged as promising tools for addressing global disparities in mental health care. However, cloud-based proprietary models raise concerns about data privacy and limited adaptability to local health care systems. In contrast, open-source LLMs offer several advantages, including enhanced data security, the ability to operate offline in resource-limited settings, and greater adaptability to non-English clinical environments. Nevertheless, their performance in psychiatric applications involving non-English language inputs remains largely unexplored.

**Objective:**

This study aimed to systematically evaluate the clinical reasoning capabilities and diagnostic accuracy of a locally deployable open-source LLM in both Korean and English psychiatric contexts.

**Methods:**

The openbuddy-mistral-7b-v13.1 model, fine-tuned from Mistral 7B to enable conversational capabilities in Korean, was selected. A total of 200 deidentified psychiatric interview notes, documented during initial assessments of emergency department patients, were randomly selected from the electronic medical records of a tertiary hospital in South Korea. The dataset included 50 cases each of schizophrenia, bipolar disorder, depressive disorder, and anxiety disorder. The model translated the Korean notes into English and was prompted to extract 5 clinically meaningful diagnostic clues and generate the 2 most likely diagnoses using both the original Korean and translated English inputs. The hallucination rate and clinical relevance of the generated clues were manually evaluated. Top-1 and top-2 diagnostic accuracy were assessed by comparing the model’s prediction with the ground truth labels. Additionally, the model’s performance on a structured diagnostic task was evaluated using the psychiatry section of the Korean Medical Licensing Examination and its English-translated version.

**Results:**

The model generated 997 clues from Korean interview notes and 1003 clues from English-translated notes. Hallucinations were more frequent with Korean input (n=301, 30.2%) than with English (n=134, 13.4%). Diagnostic relevance was also higher in English (n=429, 42.8%) compared to Korean (n=341, 34.2%). The model showed significantly higher top-1 diagnostic accuracy with English input (74.5% vs 59%; *P*<.001), while top-2 accuracy was comparable (89.5% vs 90%; *P*=.56). Across 115 questions from the medical licensing examination, the model performed better in English (n=53, 46.1%) than in Korean (n=37, 32.2%), with superior results in 7 of 11 diagnostic categories.

**Conclusions:**

This study provides an in-depth evaluation of an open-source LLM in multilingual psychiatric settings. The model’s performance varied notably by language, with English input consistently outperforming Korean. These findings highlight the importance of assessing LLMs in diverse linguistic and clinical contexts. To ensure equitable mental health artificial intelligence, further development of high-quality psychiatric datasets in underrepresented languages and culturally adapted training strategies will be essential.

## Introduction

Inequalities in access to mental health care remain a persistent global issue. This gap is particularly significant in low- and middle-income countries (LMICs), as approximately 80% of the world’s population with mental health disorders lives in LMICs [1,2]. However, this is not exclusive to LMICs. Barriers such as geographical limitations, social inequities, and inefficient allocation of human resources can contribute to insufficient access to psychiatric care [3]. Moreover, the COVID-19 pandemic has highlighted the vulnerability of the traditional mental health care model during periods of public health crisis [4,5]. These social circumstances increased interest in leveraging technology-based solutions to enhance access to mental health services [6].

Advances in artificial intelligence (AI) have emerged as promising solutions to these mental health care challenges [7]. By providing services unconstrained by time or location, AI-driven approaches can assist diverse populations regardless of social determinants [8]. AI can also facilitate prompt and appropriate mental health interventions in regions where access to mental health professionals is limited [7]. The development of large language models (LLMs) has reinforced these possibilities. LLMs have demonstrated capabilities in supporting diagnostic reasoning and assisting treatment planning in various medical fields [9]. Moreover, their conversational functionality enables applications in AI-assisted counseling and psychotherapy, such as cognitive behavioral therapy [7,10]. LLMs have also shown potential as tools for psychoeducation, responding to clinically relevant questions, and providing therapeutic advice to patients and caregivers [11].

However, adopting solutions based on LLMs has several difficulties. Cloud-based models have concerns in data security and privacy due to the transmission of data [12]. This is particularly critical in the psychiatric domain, where sensitive patient information is frequently handled during patient interviews. In contrast, open-source LLMs provide advantages such as enhanced privacy and customizability. Moreover, they provide flexibility to operate in resource-limited computing environments without requiring constant cloud access [13]. However, open-source, noncloud-dependent models also have limitations. Unlike cloud-based models, which are readily accessible via the web, open-source models require installation and technical expertise to set up. Furthermore, while cloud-based models do not impose significant hardware demands on users, open-source models can be resource-intensive, requiring substantial computational power in local environments. Despite these advantages and challenges, research on the performance of open-source LLMs in clinical settings remains limited compared with cloud-based models.

Multilingual applicability is another critical consideration in the use of LLMs in the field of psychiatry. LLMs are primarily trained on English data, with relatively less exposure to non-English data [14]. This raises questions about LLMs’ ability to deal with languages other than English in psychiatry, where subtle nuances and cultural influences are important. Although previous studies have explored the application of LLMs in non-English languages, their performance in psychiatric contexts remains underexplored [14,15].

In this study, we assessed the performance of an open-source LLM in psychiatry across two languages: Korean, as a language representing non-English languages, and English. We evaluated the reasoning ability and diagnostic accuracy of LLMs using the original Korean version and English translation of psychiatric interview notes and medical licensing examinations. With this research, we aimed to explore the potential of LLMs to improve access to psychiatric care in resource-limited settings for non-English speaking populations.

## Methods

### Ethical Considerations

This study was approved by the Institutional Review Board of Ajou University Medical Center (AJOUIRB-DB-2023‐325). The requirement for informed consent was waived due to the retrospective nature of the study, and only deidentified records were used to ensure patient confidentiality.

### Data Source

In this study, we used the electronic health records of the Ajou University Medical Center in South Korea, which were deidentified and standardized according to the Observational Medical Outcomes Partnership Common Data Model version 5.3. Records of initial psychiatric interviews from patients who visited the emergency department between March 2014 and February 2023 were extracted for analysis. For patients with multiple visits, only the initial record for each patient was included, as later records often referred to prior documentation rather than a full history.

All interview notes were initially written by psychiatry residents. To ensure training quality, consistency, and appropriate communication, residents were encouraged to use a standardized writing style and psychiatric terminology. All notes were supervised and confirmed by psychiatry professors. The interview notes consisted of two parts: present illness and assessment. The present illness section was separated and prepared for use as input to the model without further modification. For quality control, inputs with fewer than 300 tokens or more than 2000 tokens were excluded. In the assessment section, the most likely diagnosis based on the clinician’s evaluation and psychiatric history is documented and confirmed by a professor during supervision. These diagnoses were used as ground-truth labels for each input. Based on the diagnosis, all eligible psychiatric interview notes from patients with schizophrenia, major depressive disorder, bipolar disorder, or anxiety disorder were identified. From each diagnostic group, 50 cases were selected using simple random sampling without replacement, resulting in a total of 200 notes. This number was determined to balance the need for sufficient diagnostic diversity with the practical constraints of manual annotation for detailed case-level evaluation.

### Model Selection and Deployment

**Figure 1.**
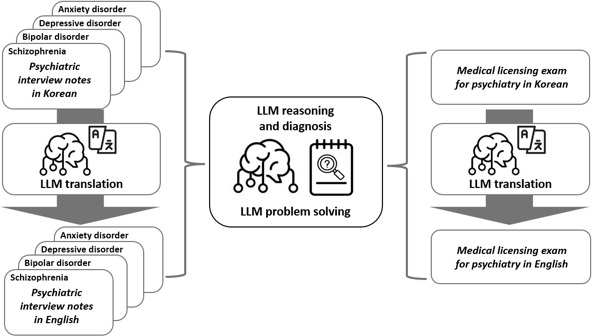
Flowchart of the study. LLM: large language model.

The study flow is illustrated in Figure 1. To assess the capability of LLMs in resource-limited environments, we explored the use of open-source LLM models with a relatively small number of parameters that can be executed locally without graphics processing unit acceleration. Among the models publicly listed on Hugging Face at the time of the study, we considered only those that officially supported Koreans. To balance diagnostic competence with hardware accessibility, we selected a 7-billion parameter model rather than a smaller 3-billion parameter alternative. We ultimately selected the openbuddy-mistral-7b-v13.1 model, a fine-tuned variant of Mistral-7B designed to enable multilingual conversation, including Korean and Chinese [16,17]. The model demonstrated the capacity to engage in coherent and consistent conversations in Korean.

To ensure compatibility with resource-limited environments, all computations in this study were performed on a local machine using a single central processing unit (2.35 GHz), without graphics processing unit acceleration. Both the translation and diagnostic inference tasks were executed under this configuration, with each process taking approximately 3 hours to complete on average. The selected model (openbuddy-mistral-7b-v13.1) was deployed using llama.cpp, an optimized C++ implementation that enables efficient execution of LLMs on central processing unit–only systems.

### Translation

The English version of the notes was created using the model itself by asking it to translate the given text into English. To ensure that the model produced the English translation as intended, we specified that the output should begin with “present illness” and imposed no additional restrictions. To evaluate the quality of the model-generated translations, all translated notes were manually reviewed by the authors (MGK, GH, and JC). Translation errors were classified into three categories: misinterpretation, omission, and addition. Each error was identified by comparing the translated output to the corresponding original Korean note. In addition to quantifying the number of translation errors, we assessed whether any error was critical enough to potentially alter the diagnostic impression. This review process was performed collaboratively through discussion to reach consensus. Any disagreements during the review were resolved through group discussion and finalized by majority consensus.

### Prompting and Output Generation

For both the Korean and English versions of each psychiatric note, the model was instructed to identify and present the 5 most important clues for diagnosis from the content of the note. The model was then instructed to answer the first and second most likely diagnoses from the four given categories: schizophrenia, bipolar disorder, depressive disorder, and anxiety disorder. All prompts were derived from the model developers’ default system prompt with minimal modification. For translation, the model was instructed to translate the given psychiatric interview note, with the only constraint that the output begin with the phrase “<Psychiatry evaluation note>” to facilitate structured parsing. For diagnostic inference, it was guided to generate 5 clues supporting its decision and choose from the four predefined diagnostic categories. The full prompts used for each task are provided in Multimedia Appendix 1. All the steps were controlled using Microsoft Guidance to ensure the validity of the outputs. Guidance is a Python library developed by Microsoft, designed to constrain text-generation models to produce outputs that adhere to specific formatting rules [18]. Using this library, users can restrict the model to select outputs from a predefined list. Through guidance, we ensured that the model selected diagnoses from the 4 diagnosis categories.

### Evaluation of Diagnostic Clues and Reasoning Ability

To evaluate the factual consistency of the model-generated clues, each clue was manually assessed by comparing it against the original source text, following the same review process applied in the translation error analysis. Clues that included information not found in the corresponding psychiatric note were classified as hallucinations. Conversely, clues that were directly verifiable within the source text were considered factually correct and were subsequently categorized into one of five mutually exclusive groups according to their diagnostic relevance. Clues that were relevant to the first and second likely diagnoses were categorized as “Diagnostic for impression #1” and “Diagnostic for impression #2,” respectively. The remaining clues, which are not directly related to the diagnosis but still possess psychiatric implications, are categorized as “Has psychiatric implications.” Clues that did not fit any of the classifications were categorized as the “No psychiatric implications” category. If clues with the same meaning appeared repeatedly, those provided later were classified into the duplicate category.

We also calculated a score representing the reasoning ability of the LLM for each note, based on the sum of the scores assigned to the clues generated from that note. The scoring system for each clue is illustrated in Figure S1 in Multimedia Appendix 2. Clues categorized as “No psychiatric implications” were assigned a score of 0 because, although they did not provide factually incorrect information, they also did not contain diagnostically useful information. Clues containing meaningful content that were categorized as “Diagnostic for impression #1,” “Diagnostic for impression #2,” and “Has psychiatric implications” were assigned scores of 3, 2, and 1, respectively. Penalties were applied to clues categorized as hallucinations or duplicates, which could reduce the quality of the output. These categories were assigned scores of −2 and −1, respectively, reflecting the greater contribution of hallucinations to inaccuracy.

### Evaluation of Diagnostic Accuracy

Diagnostic accuracy was also evaluated by comparing the ground truth with diagnostic impressions provided by the model. Top-1 and top-2 diagnostic accuracy were calculated for both the Korean and English versions of the psychiatric notes.

To further examine whether translation errors affected diagnostic performance, we divided the 200 translated notes into two groups based on translation quality. Notes with zero or one error in each of the three error types (misinterpretation, omission, and addition) were categorized as the low-error group, while the remaining notes were assigned to the high-error group. Differences in diagnostic accuracy between the Korean and English versions were also evaluated separately within the low-error and high-error groups using independent 1-tailed *t* tests. In addition, we compared diagnostic accuracy between the low-error and high-error groups within the same language condition (ie, within Korean and within English) to determine whether the number of translation errors influenced performance independently of language.

### Evaluation Using Structured Medical Examinations

To complement the analysis based on unstructured psychiatric interview notes, we conducted an additional evaluation using questions from the psychiatry section of the Korean Medical Licensing Examination (KMLE). This supplementary analysis was aimed to assess whether the model’s language-dependent performance differences persist in a structured and standardized testing context.

An open dataset, KorMedMCQA, was used to evaluate the model’s performance in the medical licensing examination. This benchmark dataset contained multiple-choice questions (MCQs) and their corresponding answers from KMLE spanning from 2012 to 2023 [16]. As the dataset included items from all medical specialties, questions related to psychiatry were manually selected by psychiatrists. In total, 115 questions were included in the analysis.

Similar to the previous analysis, the model was instructed to translate questions and corresponding choices from Korean to English. The translated versions were then used as input for subsequent evaluation, analogous to the procedure followed for the interview note analysis. Following translation, the model was prompted to select the most appropriate answer for both the original Korean and the English-translated versions of each question. Accuracy was calculated separately for each language condition.

For detailed analysis, the questions were classified into the following categories based on the *Diagnostic and Statistical Manual of Mental Disorders, Fifth Edition* (*DSM-5*): neurodevelopmental disorders, schizophrenia spectrum and other psychotic disorders, bipolar and related disorders, depressive disorders, anxiety disorders, obsessive-compulsive and related disorders, trauma- and stressor-related disorders, sleep-wake disorders, neurocognitive disorders, and personality disorders. Performance differences between the Korean and English versions were then analyzed within each diagnostic category.

### Statistical Analysis

Descriptive statistics, including means and SDs, were used to summarize the results of translation, clue classification, and reasoning score. Differences in diagnostic accuracy between Korean and English inputs were evaluated using independent 1-tailed *t* tests. For subgroup analyses, cases were divided into low-error and high-error groups based on translation quality, and diagnostic accuracy was compared using 1-tailed *t* tests within and between language conditions. For the KMLE-based evaluation, performance differences between the Korean and English versions across *DSM-5* disorder categories were analyzed using the Fisher exact test. A two-sided *P* value of <.05 was considered statistically significant.

## Results

### Translation

The types and frequencies of translation errors across ground-truth diagnostic categories are presented in Figure S2 in Multimedia Appendix 2. Across all translated texts, an average of 2.12 (SD 2.28) translation errors was identified per 250 words. Errors were further analyzed by ground truth diagnosis, and both the frequency and distribution of error types were consistent across diagnostic categories (mean errors per 250 words: schizophrenia 1.88, SD 1.25; bipolar disorder 2.10, SD 1.58; depression 1.79, SD 1.39; anxiety disorder 2.69, SD 3.84). Details of the number of translation errors are provided in Table S1 in Multimedia Appendix 2. Importantly, none of the errors were judged to be clinically significant or likely to result in a diagnostically different interpretation.

### Factual Consistency and Hallucination

In total, the model generated 997 clues from Korean interview notes and 1003 clues from notes translated into English. As shown in Table S2 in Multimedia Appendix 2, 301 (30.2%) clues derived from the Korean inputs contained information that could not be verified in the original text and were therefore classified as hallucinations. In contrast, clues generated from English inputs were more factually consistent, with the model producing only 134 (13.4%), falling into this category.

For both Korean and English notes, the model occasionally inferred that a patient had previously been diagnosed with or treated for a psychiatric condition, even though the note only described persistent symptoms without reference to any formal diagnosis or treatment history. In other cases, it produced clinically plausible but unstated clues—such as referencing sleep disturbance in a patient with depression—despite the absence of any explicit mention of such symptoms. Some hallucinations stemmed from translation errors. For example, when the model translated the Korean word “mother” as “grandmother,” the final clues included the word “grandmother,” which was absent in the original text and was therefore classified as a hallucination.

### Clinical Reasoning and Diagnostic Accuracy

The complete results of categorizing clues based on their clinical relevance are presented in Table S2 in Multimedia Appendix 2. For Korean input, 22.5% (n=224) of the responses fell into the “Diagnostic for Impression #1” category compared to 36.1% (n=362) for English input, demonstrating better results for English. When expanded to include the “Diagnostic for Impression #2” category to assess overall diagnostic capability, 42.8% (n=429) of clues from English input were relevant, compared to 34.2% (n=341) from Korean input. For the “Has psychiatric implications” category, 34.7% (n=348) of responses from English input were classified to this category compared to 28.5% (n=284) for Korean input. Factually correct but nondiagnostic clues often referred to a patient’s history of common physical illnesses, general personality traits, or aspects of their developmental background. Although these details were accurate, they were not considered directly relevant to the clinical formulation or diagnosis of the patient’s current mental disorder.

**Figure 2.**
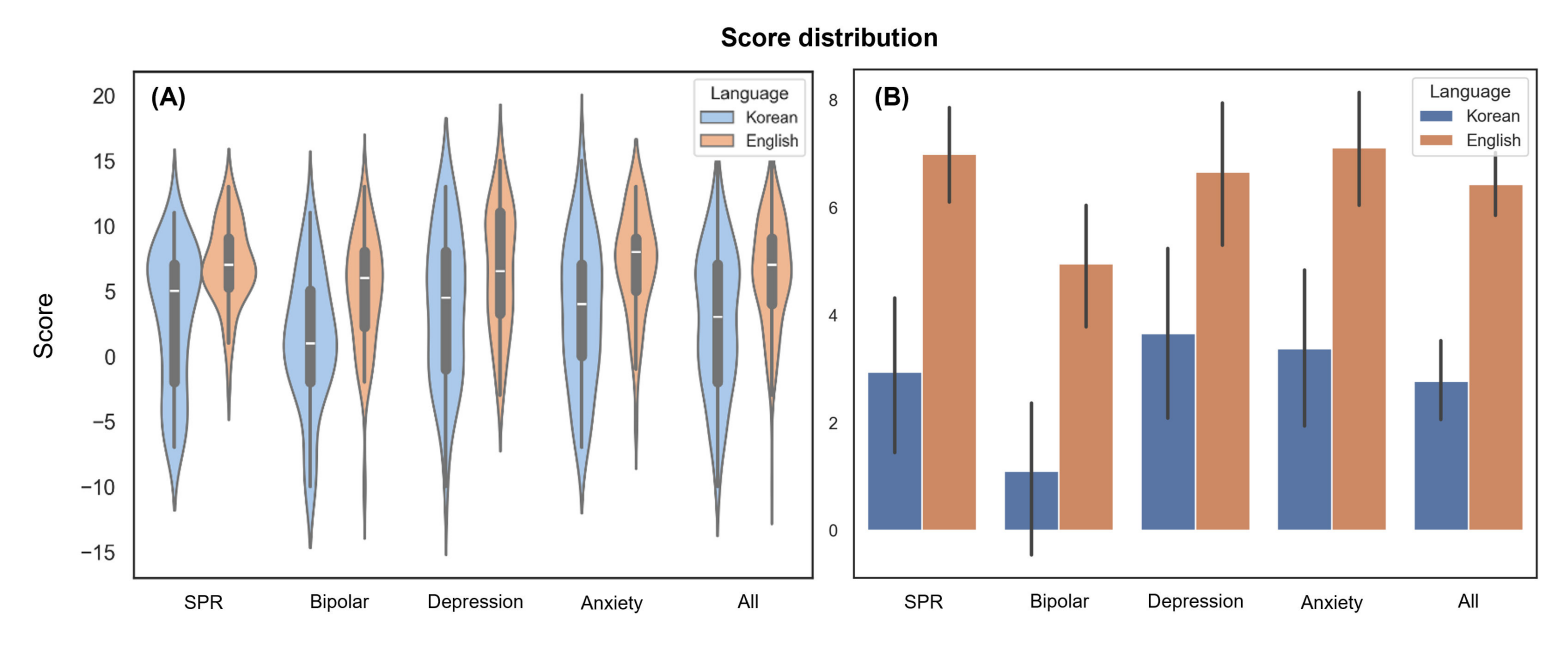
Score distribution calculated based on clues generated from Korean and English-translated psychiatric notes for each ground-truth diagnosis. (A) Violin plots showing the median and IQR. (B) Box plots displaying the mean and 95% CIs. SPR: schizophrenia.

Using the scoring system described earlier, scores were calculated based on the clues generated from psychiatric notes. English-translated input consistently showed higher median and mean scores with smaller SDs across all diagnoses (Figure 2). No outliers in score were identified across any of the four disease categories. The average reasoning score across all notes was 6.44 (SD 4.15) for English input and 2.77 (SD 5.48) for Korean input, indicating superior performance with English input. Details of the score distribution calculated based on clues are provided in Table S3 in Multimedia Appendix 2.

**Figure 3.**
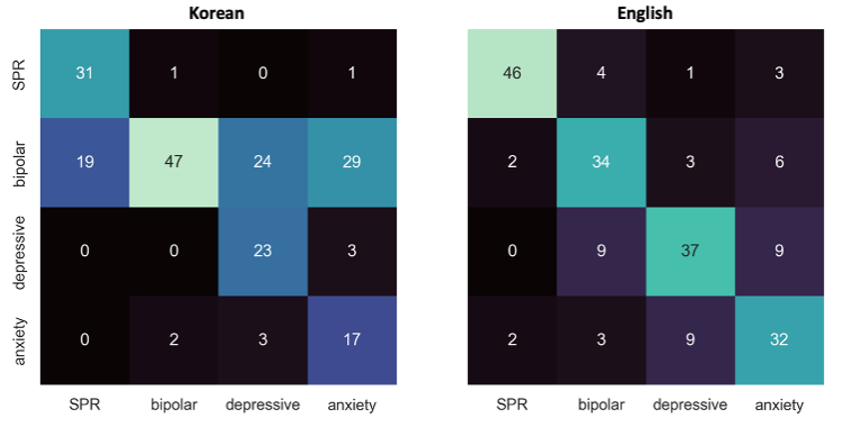
Multiclass confusion matrix describing top-1 accuracy of diagnostic impression given by the model. The x-axis represents the ground-truth diagnoses, while the y-axis represents the LLM-generated first diagnostic impression. LLM: large language model; SPR: schizophrenia.

**Figure 4.**
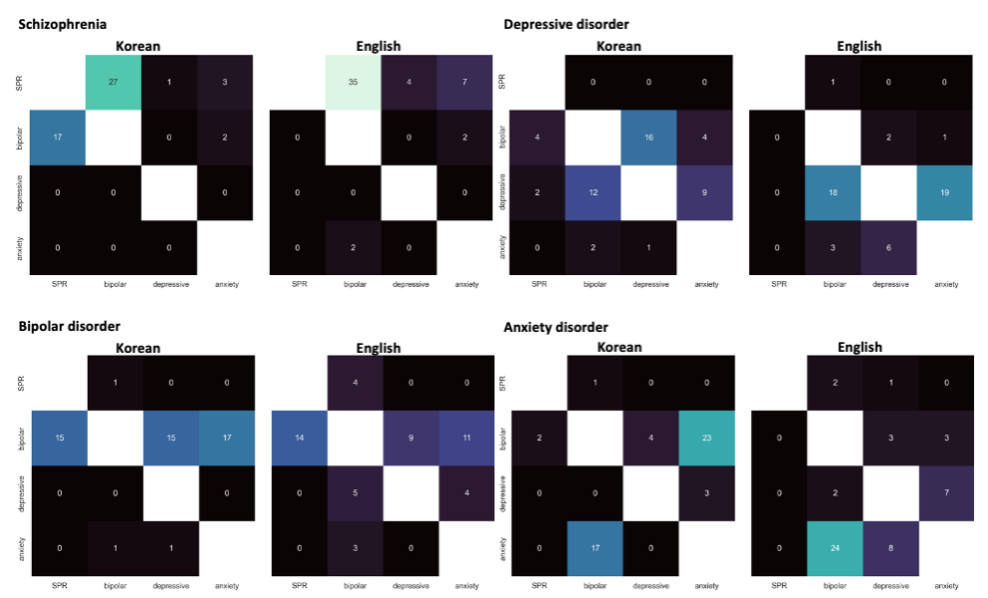
Heatmap describing the distribution of diagnostic impression pairs given by the model. The y-axis represents the first diagnostic impression, and the x-axis represents the second diagnostic impression, grouped by ground-truth diagnoses. SPR: schizophrenia.

The diagnostic impressions generated by the model from Korean and English-translated notes are presented in Tables1  2, respectively. Figure 3 illustrates the corresponding top-1 diagnostic accuracy using the confusion matrix. For all 200 notes, the Korean input achieved a top-1 accuracy of 59% (n=118), while the English-translated input performed better, with a top-1 accuracy of 74.5% (n=149). This difference was statistically significant (*t*_398_=−3.327; *P*<.001; Figure S3 in Multimedia Appendix 2), indicating that the model performed significantly better when processing English input. When analyzing the incorrect responses, a strong tendency was observed for the model to incorrectly diagnose cases as bipolar disorder when using the Korean input. Among the 50 cases of depressive disorder, the model misclassified 24 (48%) cases as bipolar disorder as the first impression. It was more pronounced for anxiety disorders, in which the model selected bipolar disorder in 29 (58%) cases. For English-translated inputs, the pattern of incorrect answers varied across the diagnosis. For schizophrenia cases, the model almost correctly identified the diagnosis. For bipolar disorder cases, the model frequently misidentified them as depressive disorder. For depressive disorder and anxiety disorder cases, the model’s usual incorrect responses were each other (ie, misclassifying depressive disorder as anxiety disorder and vice versa). The first and second diagnostic impression pairs, grouped by ground-truth diagnoses, are illustrated in Figure 4. When the first diagnostic impression provided by the model was incorrect, the model often generated the second most likely diagnosis as the correct answer. Consequently, the top-2 accuracy for Korean input increased to 90% (n=180), comparable to the English input accuracy of 89.5% (179; *t*_398_=0.164; *P*=.56; Figure S3 in Multimedia Appendix 2).

When the notes were stratified by translation quality, 63 of the 200 notes were categorized into the low-error group, and 137 into the high-error group. In both groups, top-1 diagnostic accuracy significantly improved when using English-translated inputs compared to the original Korean inputs (low-error group: *t*_124_=−2.36, *P*=.02; high-error group: *t*_272_=−2.43, *P*=.02; Figure S4 in Multimedia Appendix 2). However, no statistically significant difference in diagnostic performance was observed between the low- and high-error groups within the same language condition (Korean: *t*_198_=0.26, *P*=.80; English: *t*_198_=1.07, *P*=.29; Figure S5 in Multimedia Appendix 2).

**Table 1.** Model-generated diagnostic impressions based on Korean psychiatric interview notes.

Top-2 diagnosis generated by the model		Ground-truth diagnosis			
		Schizophrenia (n=50)	Bipolar (n=50)	Depression (n=50)	Anxiety (n=50)
Schizophrenia (first)					
Bipolar (second)		27	1	0	1
Depression (second)		1	0	0	0
Anxiety (second)		3	0	0	0
Total		31	1	0	1
Bipolar (first)					
Schizophrenia (second)		17	15	4	2
Depression (second)		0	15	16	4
Anxiety (second)		2	17	4	23
Total		19	47	24	29
Depression (first)					
Schizophrenia (second)		0	0	2	0
Bipolar (second)		0	0	12	0
Anxiety (second)		0	0	9	3
Total		0	0	23	3
Anxiety (first)					
Schizophrenia (second)		0	0	0	0
Bipolar (second)		0	1	2	17
Depression (second)		0	1	1	0
Total		0	2	3	17

**Table 2.** Model-generated diagnostic impressions based on English-translated psychiatric interview notes.

Top-2 diagnosis generated by the model		Ground-truth diagnosis			
		Schizophrenia (n=50)	Bipolar (n=50)	Depression (n=50)	Anxiety (n=50)
Schizophrenia (first)					
Bipolar (second)		35	4	1	2
Depression (second)		4	0	0	1
Anxiety (second)		7	0	0	0
Total		46	4	1	3
Bipolar (first)					
Schizophrenia (second)		0	14	0	0
Depression (second)		0	9	2	3
Anxiety (second)		2	11	1	3
Total		2	34	3	6
Depression (first)					
Schizophrenia (second)		0	0	0	0
Bipolar (second)		0	5	18	2
Anxiety (second)		0	4	19	7
Total		0	9	37	9
Anxiety (first)					
Schizophrenia (second)		0	0	0	0
Bipolar (second)		2	3	3	24
Depression (second)		0	0	6	8
Total		2	3	9	32

### Performance in Structured Medical Examinations

The performance of the LLM on the KMLE and its English-translated version is described in Table S4 in Multimedia Appendix 2. The model demonstrated better overall accuracy in English, answering 53 (46.1%) of 115 total questions correctly, compared to 37 (32.2%) correct answers in Korean. When analyzed by the topics of the questions, the result was better in English for 7 out of 11 categories. The difference was especially marked in obsessive-compulsive and related disorders, and bipolar and related disorders. However, statistical significance only existed for the bipolar and related disorders categories. Korean inputs demonstrated better performance only in the neurocognitive disorder category, but the difference was marginal. The number of questions included in each category of the exam and the corresponding number of correct answers provided by the model are detailed in Table S4 in Multimedia Appendix 2.

## Discussion

### Principal Findings

This study evaluated the diagnostic performance and reasoning quality of a small-scale, open-source LLM in psychiatry using psychiatric interview notes written in Korean and their English translations. A multilingual conversational model was prompted to extract diagnostic clues and generate first and second likely psychiatric diagnoses for each case. When processing Korean input, the model generated a significantly higher rate of hallucinations compared to English input (30.2% vs 13.4%). The model also produced fewer diagnostically relevant clues with Korean input compared to English input (34.2% vs 42.8%). Similarly, a lower proportion of clues from Korean input was categorized as having general psychiatric implications compared to English input (28.5% vs 34.7%). Aligned with these findings, the model demonstrated significantly lower top-1 diagnostic accuracy for Korean input than English input (59.0% vs 74.5%). These discrepancies persisted regardless of translation quality. Notably, Korean input also showed a characteristic misclassification pattern, with the model frequently overpredicting bipolar disorder in cases of depression and anxiety. While top-2 accuracy was comparable in both languages, structured evaluation using the KMLE confirmed similar trends, with higher accuracy on English-translated questions across most diagnostic categories.

### Comparison to Prior Work

Our findings suggest that the performance of open-source LLMs in psychiatric contexts may vary depending on the language used, particularly in resource-limited settings. Since the training data for most LLMs is predominantly in English, there have been concerns regarding their capability in non-English contexts [19]. With the emergence of translation and multilingual input capabilities in LLMs with larger parameters, many of the challenges faced by non-English speakers appeared to be resolved. In previous studies, GPT-4 demonstrated good performance on medical examinations in several non-English contexts [20]. However, studies comparing ChatGPT’s performance in the original language with the English-translated version have consistently shown better results for the English-translated version across various languages, including Chinese, Spanish, and Persian [14,21]. One study evaluated the performance of ChatGPT in Chinese and English contexts in ophthalmology, specifically assessing its ability to diagnose retinal vascular disease using fundus fluorescein angiography reports [19]. In that study, ChatGPT performed better diagnostic and inference capabilities when prompted with English. This finding aligns with this study, indicating that general multilingual capabilities do not necessarily mean proficiency in specialized domains. Furthermore, this study indicates that this disparity may be even more pronounced for smaller LLMs operating in local environments, underscoring the importance of targeted efforts to address this issue.

The differential performance of LLMs across languages observed in this study suggests potential sociodemographic biases in LLM outputs, particularly in multilingual and culturally diverse settings. Previous studies suggest that LLM outputs may overrepresent the Western population and fail to reflect unique demographic characteristics, even when clinically necessary [22,23]. For example, patients with mood disorders tend to present with somatic complaints rather than explicit emotional distress or cognitive symptoms in East Asian cultures, including Korea. This difference may increase hallucination rates and reduce diagnostic accuracy when applying LLMs trained on English texts to non-English data. Such disparities raise ethical and clinical concerns, as reliance on such models in psychiatric assessments may reinforce existing health care inequities, contrary to the expectation that LLMs could enhance accessibility and improve health equity [24]. Addressing these issues will be necessary to ensure the effective use of LLMs in psychiatric settings across diverse cultural and linguistic contexts.

As our research has shown, using an English translation may be one way to improve the quality of the output. However, subtle nuances and elements in a cultural context can be difficult to translate and may lose their unique meaning during translation [25]. For example, Hwabyung is a psychological term used in Korea to describe a condition that arises when an individual experiences intense anger due to perceived injustice but is unable to express or confront these emotions due to social constraints, leading to emotional suppression [26]. It is typically accompanied by somatic symptoms such as chest tightness or a feeling of heat rising from the gut. Hwabyung lacks a direct equivalent in Western diagnostic frameworks; its translation may not fully capture its cultural and clinical significance, leading to potential misinterpretation in LLM-based psychiatric applications. Furthermore, the extra step for translation can itself be a barrier for users who are not fluent in English [27]. They may encounter difficulties in understanding the translated output and lack confidence in whether the translated input accurately reflects their intentions [28]. Therefore, incorporating translation as an intermediate process is not an inherent solution when considering the use of LLMs to improve mental health care accessibility for the general population.

Fine-tuning for specific purposes or using retrieval-augmented generation (RAG) with targeted data written in the specific language can be another strategy to enhance the performance of LLMs [13,29]. For example, fine-tuning an LLM using patient education materials written in Korean could enhance its ability to generate accurate and contextually appropriate responses when explaining various mental health conditions to patients. Adopting RAG with the Korean version of official diagnostic criteria or psychiatric textbooks in Korean could help minimize hallucinations and improve the use of appropriate medical terminology and culturally relevant expressions. Additionally, integrating RAG with electronic medical records could enable the model to generate context-aware, personalized responses informed by detailed patient data from psychiatric notes. However, high-quality psychiatric datasets should be prepared to apply these approaches. These datasets should contain accurate clinical interpretations of psychiatric terminology, appropriate use of nuanced symptom descriptions, and consideration of cultural contexts [25]. But unfortunately, building such a system can be more difficult in the regions where AI-based assistance could be most beneficial due to limited resources [30]. Continuous collaboration between clinicians, AI researchers, and policy makers is essential to address these challenges.

Despite several challenges, open-source LLMs have advantages that can make them particularly valuable in psychiatry. They can be deployed locally, which ensures that sensitive patient data never leaves the institution [31]. This is critical in psychiatry, where confidentiality and privacy are crucial during treatment. In addition to confidentiality, flexibility is a distinguishing feature of open-source models. They can be customized to handle specific clinical circumstances, regional characteristics, and variations in health care systems [32]. Open-source models can also serve as cost-effective tools for resource-limited settings. Their ability to operate on local devices without requiring cloud infrastructure makes them accessible to underserved populations where internet connectivity is inconsistent or unavailable. Furthermore, they can also support primary care providers or community health workers in delivering basic mental health care, bridging gaps in services where specialized psychiatric care is scarce [6,33]. These advantages of open-source LLMs underscore the importance of continued exploration of their use in psychiatry.

### Strengths and Limitations

This study has several key strengths. First, to our knowledge, this is the first study in psychiatry to evaluate the performance of an LLM on non-English clinical notes by directly comparing it with its performance on English-translated counterparts. Despite the critical importance of cultural and linguistic context in psychiatric evaluation, evidence in this area remains limited. Our findings underscore the importance of evaluating the performance of LLMs across multiple languages and models to ensure their safe and equitable application in diverse psychiatric settings. Second, unlike prior studies that rely on MCQs, we evaluated model performance using actual psychiatric interview notes. Although MCQs offer standardized formats, they do not fully capture the complexity of the clinical context. By leveraging real-world documentation that reflects diverse patient presentations, this study provides a more clinically meaningful evaluation of LLM capabilities. Third, rather than merely evaluating diagnostic labels, we conducted a comprehensive and systematic analysis of the model’s reasoning by examining the diagnostic clues it generated. This approach enabled us to assess the association between the model’s clinical reasoning and its final decisions, facilitating a structured comparison and enhancing the explainability of the observed differences.

This study has several limitations. First, the comparison of performance differences between languages was primarily conducted for Korean and English. Many languages with fewer speakers than Korean may have limited exposure during the training process of LLMs. Future studies should evaluate LLM performance across a broader range of languages to provide a more comprehensive understanding of their applicability in psychiatry. Second, we designed and applied a scoring system specifically developed for this study to evaluate the results. Since there is no gold standard framework for evaluating LLM outputs in their psychiatric competence, it was necessary to invent our own scoring system. To address potential bias in our scoring system, we conducted additional experiments using structured medical examinations and observed consistent results. Further research is needed to develop methods to objectively assess the performance of LLMs in psychiatry. Third, the translation step in this study may have introduced systematic biases that could affect model performance. Although we confirmed that LLM-generated translations did not alter the diagnosis of original texts, translated texts may not have fully preserved the original intent or nuanced expressions specific to mental health. Moreover, culturally embedded or idiomatic phrases might have been translated into more explicit or conventional terms in English, potentially enhancing model performance. Further studies comparing human-translated and LLM-translated texts are warranted to better characterize the impact of translation on diagnostic inference. Fourth, the psychiatric interview notes used in this study were obtained from a single Korean medical center, which may limit the generalizability of our findings. Variations in psychiatric terminology, documentation practices, and linguistic expression across institutions and regions may affect the model’s performance and its applicability to broader clinical contexts. Nevertheless, we advanced beyond previous studies, which primarily relied on hypothetical data or clinical vignettes with cloud-based LLMs [11]. Leveraging the advantages of open-source LLMs, we used real-world clinical interviews from actual patients to address more realistic scenarios while ensuring the protection of privacy. Despite several limitations, this study highlights both the challenges and opportunities associated with LLM applications in psychiatry. We hope that this study will serve as a supporting step for future research.

### Conclusions

In this study, we evaluated the potential of open-source LLMs to enhance mental health support in non-English contexts and resource-limited settings. Our findings indicate that clinical reasoning ability and diagnostic accuracy may be higher in English compared to Korean within psychiatric contexts. The strengths of open-source LLMs, including privacy, customizability, and accessibility, make them promising tools for expanding mental health support. Further efforts are needed to develop LLMs with robust multilingual psychiatric capabilities.

## Supplementary material

10.2196/69857 Multimedia Appendix 1 Prompts used for translation, diagnostic inference, and clue generation.

10.2196/69857 Multimedia Appendix 2 Supplementary figures and tables.

## Abbreviations

AI: artificial intelligence
*DSM-5*: *Diagnostic and Statistical Manual of Mental Disorders, Fifth Edition*
KMLE: Korean Medical Licensing Examination
LLM: large language model
LMIC: low- and middle-income country
MCQ: multiple-choice question
RAG: retrieval-augmented generation
