# Supplementary material for: Performance of Open-Source Large Language Models in Psychiatry: Usability Study Through Comparative Analysis of Non-English Records and English Translations
Source: J Med Internet Res. 2025 Aug 18;27:e69857. doi: 10.2196/69857 (PMC12360790; doi:10.2196/69857)
Supplement: Multimedia Appendix 2 [file jmir-v27-e69857-s002.docx]

**Figure S1.** Flowchart for the classification of clues generated from psychiatric notes.


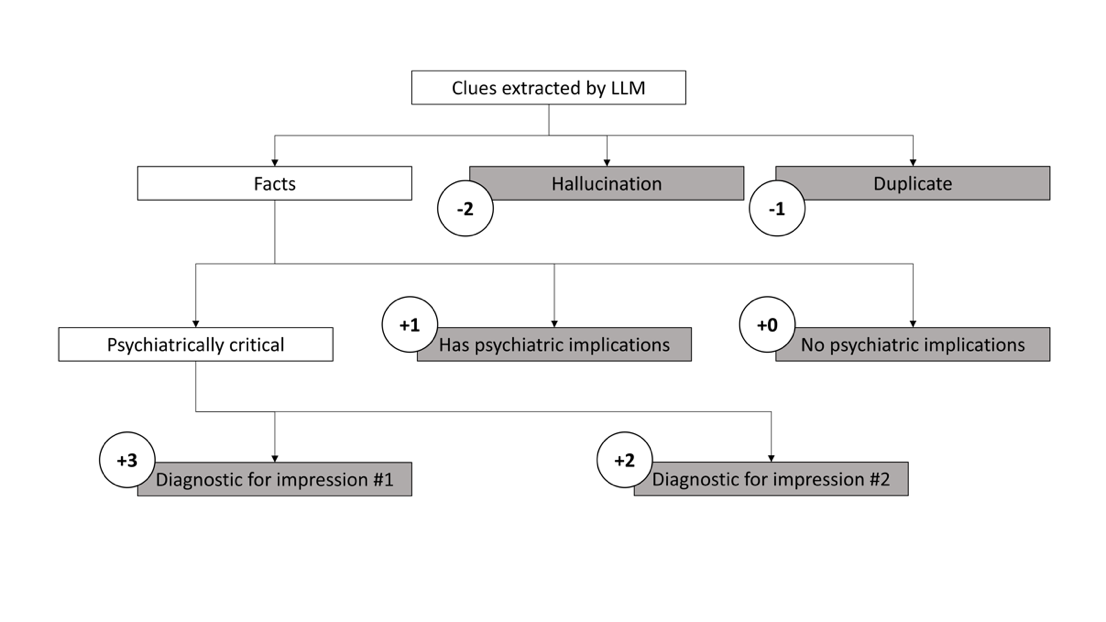


**Figure S2.** The types and frequencies of translation errors across ground-truth diagnostic categories.

**
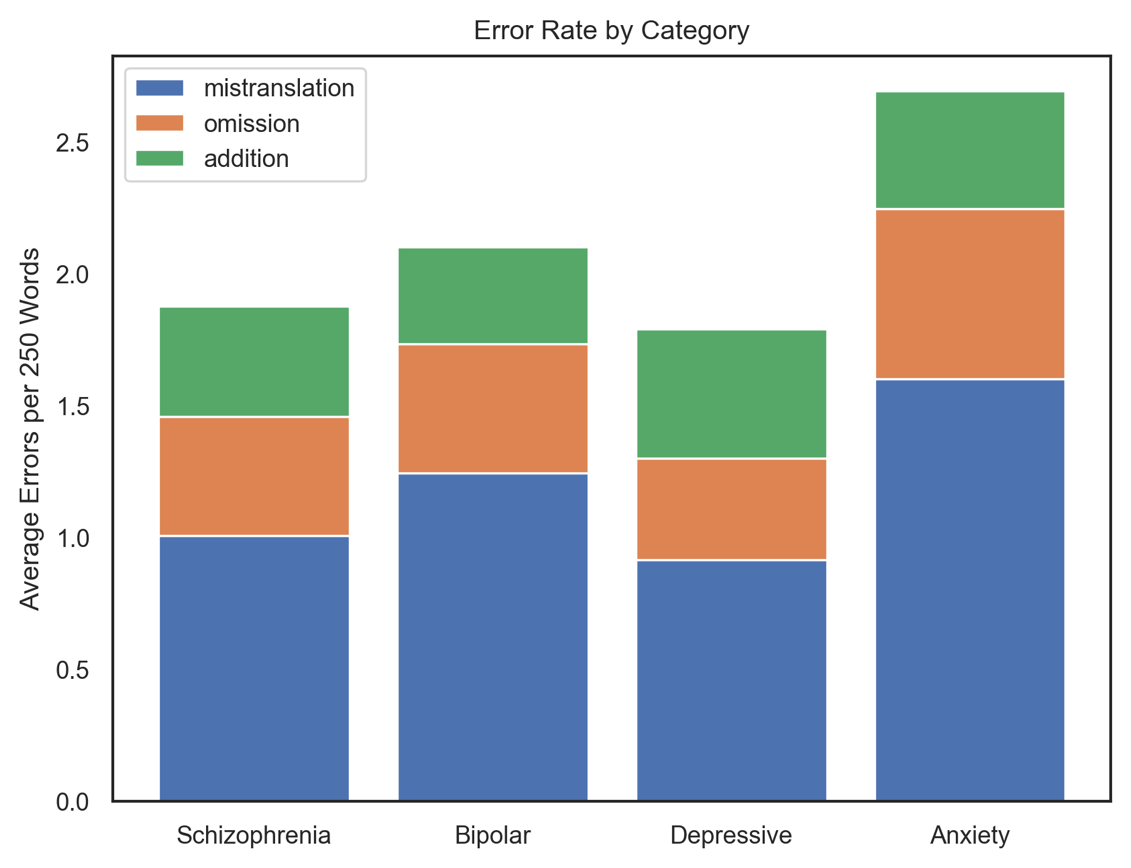
**

**Figure S3.** Top-1 and top-2 diagnostic accuracy of the model with Korean and English-translated inputs.

**
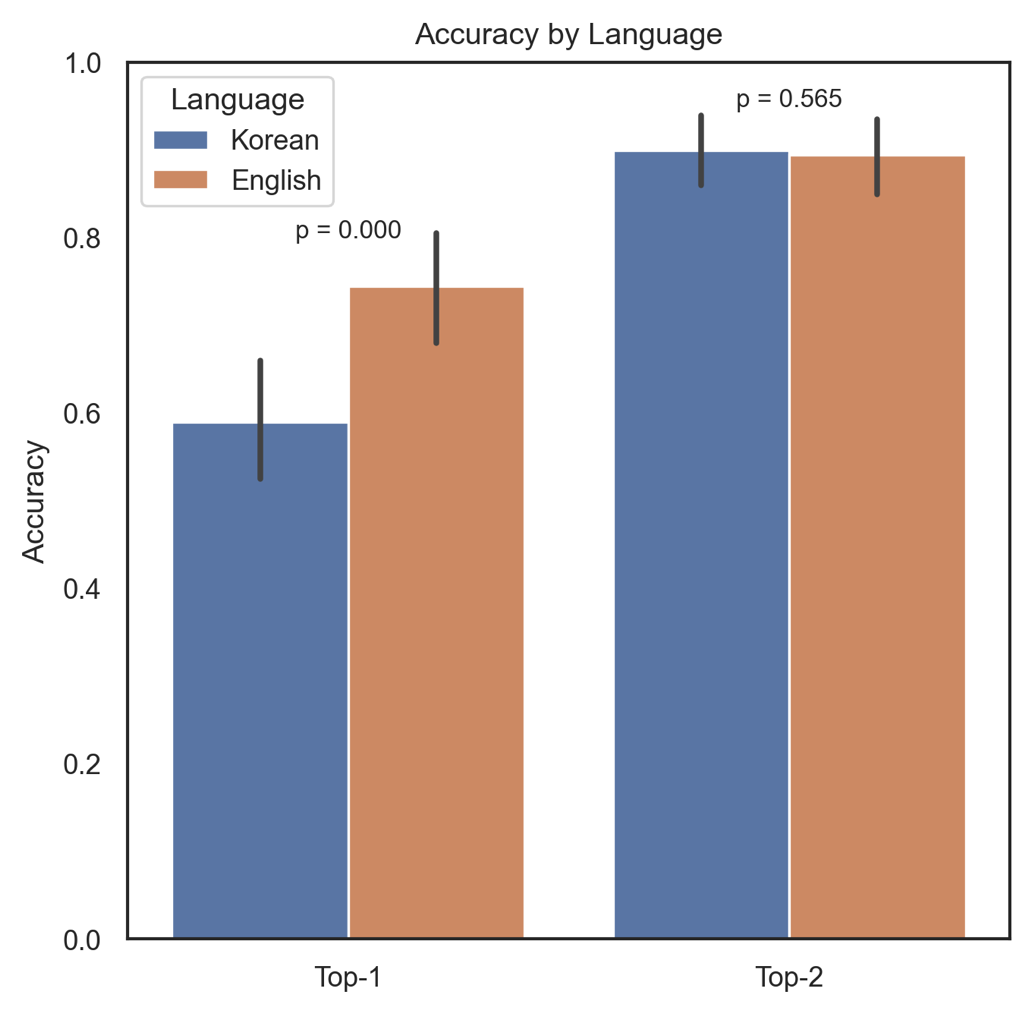
**

**Figure S4.** Top-1 accuracy of the model with Korean and English-translated inputs stratified by translation error rate.

**
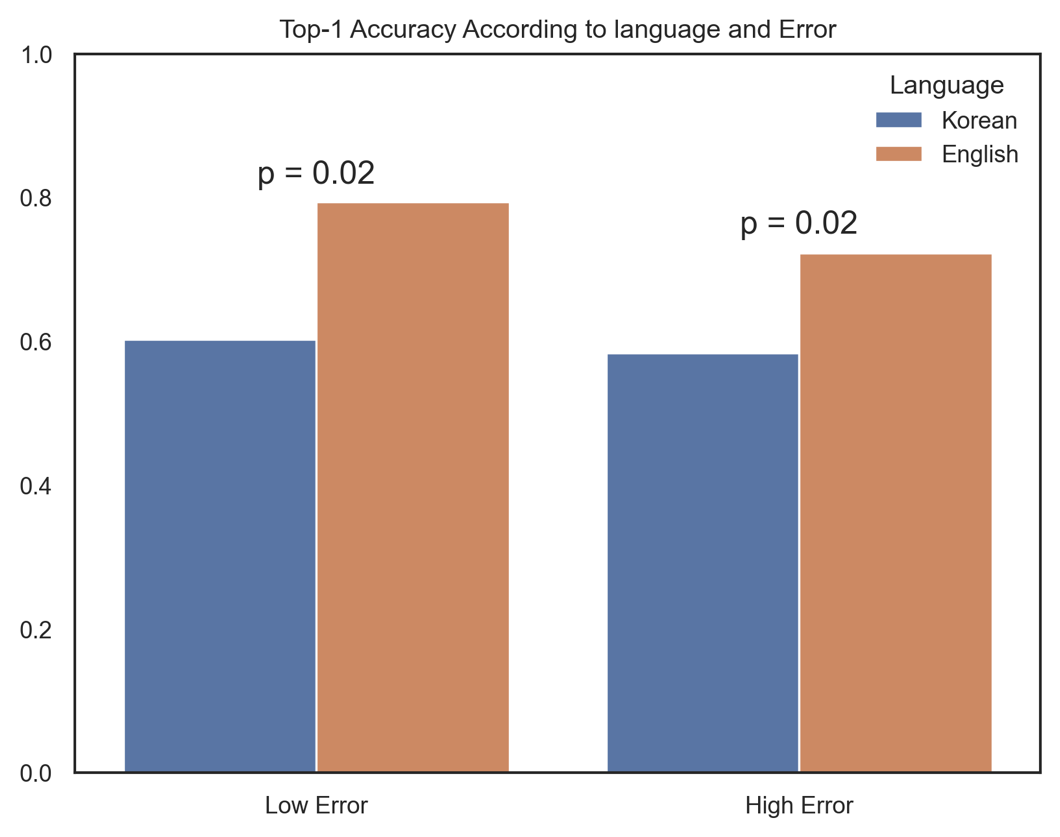
**

**Figure S5.** Top-1 accuracy of the model with low-error and high-error inputs stratified by language.

**
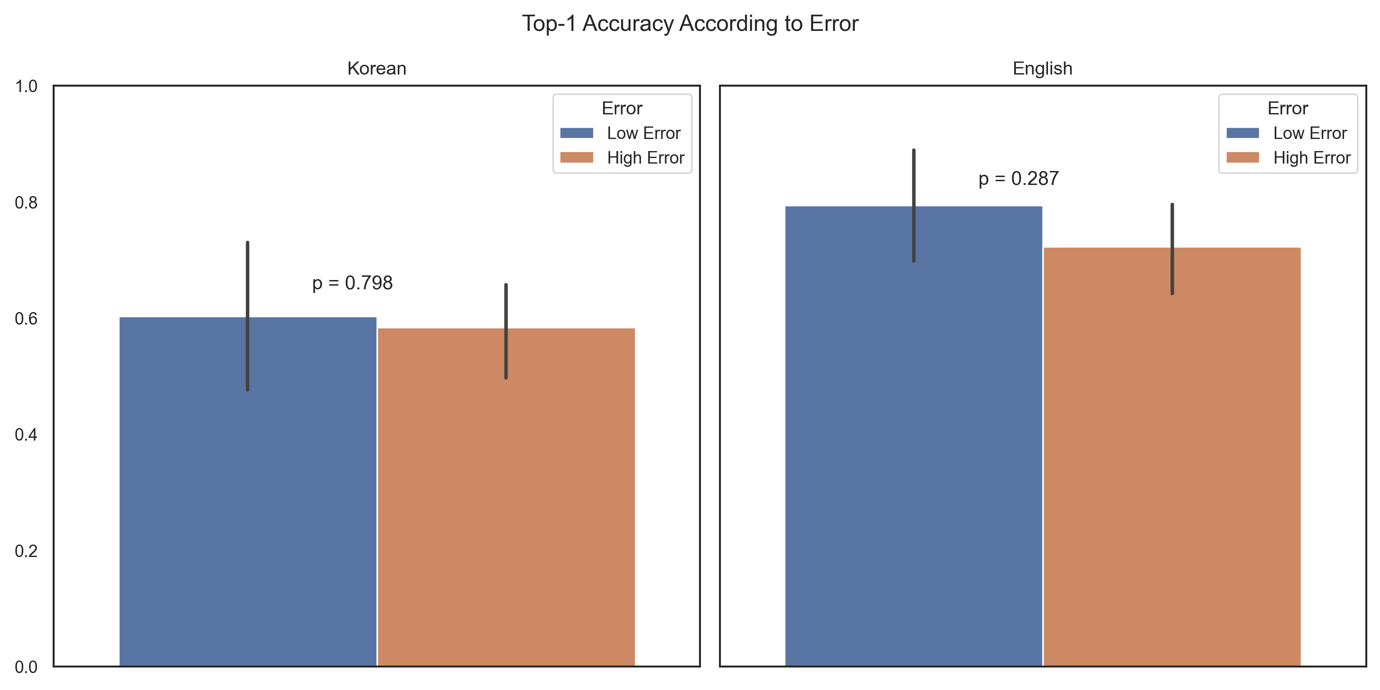
**

**Table S1**. The number of translation errors per 250 words across ground-truth diagnostic categories.

| Ground-truth | Mean | SD | Minimum | 25th-percentile | 50th-percentile | 75th-percentile | Maximum |
| --- | --- | --- | --- | --- | --- | --- | --- |
| SPR | 2.69 | 3.84 | 0.00 | 0.98 | 1.89 | 2.65 | 24.04 |
| Bipolar | 2.10 | 1.58 | 0.00 | 0.97 | 1.72 | 3.02 | 7.81 |
| Depressive | 1.79 | 1.39 | 0.00 | 1.00 | 1.56 | 2.43 | 7.35 |
| Anxiety | 1.88 | 1.25 | 0.00 | 1.08 | 1.69 | 2.50 | 6.43 |

SD: Standard deviation; SPR: Schizophrenia

**Table S2**. The number of clues classified into each category based on clinical relevance.

| **Category** | **Diagnostic impression #1** | **Diagnostic impression #2** | **Has psychiatric implication** | **No psychiatric implication** | **Duplicate** | **Hallucination** | **Total** |
| --- | --- | --- | --- | --- | --- | --- | --- |
| Korean | 224 | 117 | 284 | 37 | 34 | 301 | 997 |
| English | 362 | 67 | 348 | 79 | 13 | 134 | 1003 |

**Table S3**. Score distribution calculated based on clues generated from Korean and English psychiatric notes for each ground-truth diagnosis.

| **From original Korean notes** | | | | | | | | |
| --- | --- | --- | --- | --- | --- | --- | --- | --- |
| Ground-truth | | Mean | SD | Minimum | 25th-percentile | 50th-percentile | 75th-percentile | Maximum |
| SPR | | 2.94 | 5.29 | -7 | -2 | 5 | 7 | 11 |
| Bipolar | | 1.10 | 5.14 | -10 | -2 | 1 | 5 | 11 |
| Depressive | | 3.66 | 5.73 | -10 | -1 | 4.5 | 8 | 13 |
| Anxiety | | 3.38 | 5.52 | -7 | 0 | 4 | 7 | 15 |
| Total | | 2.77 | 5.48 | -10 | -2 | 3 | 7 | 15 |
| **From English-translated notes** | | | | | | | | |
| Ground-truth | | Mean | SD | Minimum | 25th-percentile | 50th-percentile | 75th-percentile | Maximum |
| SPR | | 7.00 | 3.16 | -2 | 5.25 | 7 | 9 | 13 |
| Bipolar | | 7.12 | 3.98 | -5 | 5 | 8 | 9 | 13 |
| Depressive | | 4.96 | 4.37 | -10 | 2.25 | 6 | 8 | 13 |
| Anxiety | | 6.66 | 4.70 | -3 | 3.25 | 6.5 | 11 | 15 |
| Total | 6.44 | | 4.15 | -10 | 4 | 7 | 9 | 15 |

SD: Standard deviation; SPR: Schizophrenia

**Table S4**. Model performance on the medical licensing exam in Korean and English.

| **Categories (Number of Questions)** | **Number of Correct Answers** | | **P-value** |
| --- | --- | --- | --- |
|  | **Korean Version** | **English Version** |  |
| Neurodevelopmental disorders (22) | 7 | 10 | 0.27 |
| Schizophrenia spectrum and other psychotic disorders (9) | 3 | 4 | 0.5 |
| Bipolar and related disorders (10) | 1 | 6 | 0.03 |
| Depressive disorders (11) | 4 | 4 | 0.67 |
| Anxiety disorders (10) | 4 | 4 | 0.68 |
| Obsessive-compulsive and related disorders (5) | 1 | 4 | 0.1 |
| Trauma- and stressor-related disorders (8) | 3 | 5 | 0.31 |
| Somatic symptom and related disorders (10) | 2 | 4 | 0.31 |
| Sleep–wake disorders (5) | 3 | 3 | 0.74 |
| Neurocognitive disorders (18) | 7 | 6 | 0.76 |
| Personality disorders (7) | 2 | 3 | 0.5 |
